# The Hypodermic Syringe

**Published:** 1885-06-20

**Authors:** J. S. Geigley

**Affiliations:** Lewiston, Ill.


### THE HYPODERMIC SYRINGE.

BY J. S. GEIGLEY, M. D., Lewiston, Ill.

In Dr. Bumstead's communication to the Clinical Society, in the last number of the *Monthly*, he made some observations on hypodermic injections, and, as this is a matter that is not very frequently discussed, I think I may be pardoned for delivering myself of a few thoughts on the subject. I have often been surprised at the ignorance or carelessness manifested in the use of this little instrument by many physicians; men, too, who were able and acute in other things, but who seemed to have failed to become thoroughly acquainted with an instrument that is so potent for good, when judiciously used. For my own part, I should feel utterly helpless without a good hypodermic syringe, in the treatment of some of the more malignant malarial affections which we often meet along the river bottoms, not to say anything of the innumerable *algias* over which the hypodermic syringe exerts such a magical influence.

Now, what are the requirements for success with the hypodermic method? First of all, we must have a good instrument, the different parts of which should fit so accurately as not to admit of the loss of a single drop of the solution, except through the needle. The piston should fit closely, but not tightly. A good test for a syringe is to withdraw the piston while stopping the opening at the other end with the finger. If the piston rebounds on being released, the instrument can be relied on. Now, next comes the needle. A good needle is everything, while a poor one is an abomination. If I were given my choice between a good syringe with a poor needle and a poor syringe with a good needle, I should choose the latter, every time, for I should be much more confident of getting my solution in the right place with it. The needle should be of good size, and not too long. A large needle gives no more pain than a small one, and does not become clogged so easily. The point should be perfectly sharp, with no rough edge, and should have the very slightest bend in the direction of the bevel. An elegant needle has lately been placed on the market, under the name of the "re-inforced hypodermic needle." Now, as a good instrument might be rendered worthless in a short time by not having the proper care, perhaps a few words on that subject would not be out of place.

The piston should be occasionally removed from the barrel, and the packing thoroughly cleaned and oiled. It is always well enough to keep a drop or two of water above the piston. This keeps the leather from shrinking if the instrument is not used for several days. Always after using the syringe the needle should be thoroughly cleansed. This is important, for two reasons—first, it preserves the potency of the opening and keeps the needle in good order; second, it might prevent infecting some one with a contagious disease. A little dilute acid first, and water afterward, drawn into the syringe, will accomplish the first and prevent the second. If a needle should become clogged so that the obstruction cannot be removed with the wire, or by soaking in dilute acid, it will be found to clear instantly if held in the flame of a spirit lamp, or even a match. True, this will draw the temper of the needle, but it can be pretty well restored by plunging it in a glass of cold water, while at a red heat.

Now, as to the points of election for the insertion of the needle: For all ordinary drugs, such as solutions of morphia, atropia, apomorphia, pilocarpine, etc., the backs of the arms, anywhere from the wrists to the shoulders, are preferable on account of being less sensitive and there being fewer vessels to run the risk of puncture. The same is true of the lower limbs, but when irritating injections, such as acid solution of quinine, chloral, or ergot, are to be used, they will be found to give the least trouble if injected in the buttocks, or on the backs of the shoulders. In hypersensitive persons, some kind of a local anæsthetic will be found useful. I have found that a small piece of ice pressed on the skin for a short time before making the punctures will answer admirably. Another point worth remembering, is, not to use too much strength in picking up the skin in which to make your puncture; for, in an individual with a delicate skin, an unsightly discoloration would be apt to follow, which would be annoying to the patient and embarrassing to the physician.

Now, a few words in regard to the solution usually employed for hypodermic use, and I am done. These should, as a rule, be prepared as used; the compressed hypodermic tablets now on the market enable us to do this with little loss of time and perfect accuracy. If the solution is heated in a spoon over a lamp, before being drawn into the syringe, it will be rendered perfectly aseptic, and, as the solution is more perfect, it is less liable to create local disturbance.

In regard to the hypodermic use of the various drugs, I shall only refer to one specialty, and that is, quinine. In my opinion, five grains, in proper solution, of this drug, given hypodermically, will do more toward breaking up an intermittent or a remittent fever, than twenty grains will do if given by the mouth. Then, too, the cerebral disturbance will not be so great. There is a great saving of the drug. And we will not be depending upon a disordered stomach for the absorption of our quinine.

The solution I use is this:

| | |
|---|---|
| ℞. Quiniæ sulph | 5 grains |
| Acid. tartaric | 2½ grains |
| Aqua | q. s. ad. 1 drachm. |

This is to be boiled in a test tube over a spirit lamp, until perfectly clear. It will not precipitate on cooling, and being quite dilute, will not cause abscess, and but very little pain. To break up an intermittent, I usually inject all of the solution, half in each shoulder, two hours before the expected chill. I treat neuralgia of malarial origin in the same way, only adding a quarter of a grain of morphia to the solution.—*Peoria Medical Monthly.*

